# Supplementary material for: Quantifying complexity in DNA structures with high resolution Atomic Force Microscopy
Source: Nat Commun. 2025 Jul 1;16:5482. doi: 10.1038/s41467-025-60559-x (PMC12216981; doi:10.1038/s41467-025-60559-x)
Supplement: Supplementary file 2 — Reporting Summary [file 41467_2025_60559_MOESM2_ESM.pdf]

nature portfolio

Corresponding author(s): Sean D. Colloms, and Alice L. B. Pyne.

Last updated by author(s): 01/05/2024

Reporting Summary

Nature Portfolio wishes to improve the reproducibility of the work that we publish. This form provides structure for consistency and transparency in reporting. For further information on Nature Portfolio policies, see our Editorial Policies and the Editorial Policy Checklist.

Statistics

For all statistical analyses, confirm that the following items are present in the figure legend, table legend, main text, or Methods section.

n/a	Confirmed	
☐	☒	The exact sample size (n) for each experimental group/condition, given as a discrete number and unit of measurement
☐	☒	A statement on whether measurements were taken from distinct samples or whether the same sample was measured repeatedly
☐	☒	The statistical test(s) used AND whether they are one- or two-sided<br>*Only common tests should be described solely by name; describe more complex techniques in the Methods section.*
☒	☐	A description of all covariates tested
☒	☐	A description of any assumptions or corrections, such as tests of normality and adjustment for multiple comparisons
☐	☒	A full description of the statistical parameters including central tendency (e.g. means) or other basic estimates (e.g. regression coefficient) AND variation (e.g. standard deviation) or associated estimates of uncertainty (e.g. confidence intervals)
☒	☐	For null hypothesis testing, the test statistic (e.g. F, t, r) with confidence intervals, effect sizes, degrees of freedom and P value noted<br>*Give P values as exact values whenever suitable.*
☒	☐	For Bayesian analysis, information on the choice of priors and Markov chain Monte Carlo settings
☒	☐	For hierarchical and complex designs, identification of the appropriate level for tests and full reporting of outcomes
☒	☐	Estimates of effect sizes (e.g. Cohen's d, Pearson's r), indicating how they were calculated

Our web collection on statistics for biologists contains articles on many of the points above.

Software and code

Policy information about availability of computer code

Data collection	AFM data was collected on Bruker Multimode 8 and FastScan Dimension XR systems, using their software Nanoscope (v10).
Data analysis	AFM data was analysed using Python (v3.10) scripting available at https://github.com/AFM-SPM/TopoStats (v2.3.0) DOI: 10.15131/shef.data.22633528.v2. Additional python analysis scripts are available with the complete datasets from this study via Figshare DOI: 10.15131/shef.data.27143238.

For manuscripts utilizing custom algorithms or software that are central to the research but not yet described in published literature, software must be made available to editors and reviewers. We strongly encourage code deposition in a community repository (e.g. GitHub). See the Nature Portfolio guidelines for submitting code & software for further information.

Data

Policy information about availability of data

All manuscripts must include a data availability statement. This statement should provide the following information, where applicable:

- Accession codes, unique identifiers, or web links for publicly available datasets
- A description of any restrictions on data availability
- For clinical datasets or third party data, please ensure that the statement adheres to our policy

All data used in this publication, and plotting scripts are publicly available on Figshare with DOI https://doi.org/10.15131/shef.data.27143238. The data for each figure are also provided in the Source Data file.

Research involving human participants, their data, or biological material

Policy information about studies with human participants or human data. See also policy information about sex, gender (identity/presentation), and sexual orientation and race, ethnicity and racism.

Reporting on sex and gender	N/A
Reporting on race, ethnicity, or other socially relevant groupings	N/A
Population characteristics	N/A
Recruitment	N/A
Ethics oversight	N/A

Note that full information on the approval of the study protocol must also be provided in the manuscript.

Field-specific reporting

Please select the one below that is the best fit for your research. If you are not sure, read the appropriate sections before making your selection.

☒ Life sciences ☐ Behavioural & social sciences ☐ Ecological, evolutionary & environmental sciences

For a reference copy of the document with all sections, see nature.com/documents/nr-reporting-summary-flat.pdf

Life sciences study design

All studies must disclose on these points even when the disclosure is negative.

Sample size	For AFM: sample sizes were obtained due to reproducibility of the previous experiment. Each experiment was repeated multiple times with the same conditions and showed the same trend when analysed using our automated pipeline. High resolution images are difficult to perform and more time consuming therefore less repeats were obtained for these measurements. However, multiple molecules from at least two samples were analysed.<br>For simulation data: the simulation data was parameterised from the AFM data, 4 different conditions were run, and to avoid duplication only the last structures from the equilibrated and adsorbed simulations were used to avoid duplication of data.
Data exclusions	Low resolution AFM data of resolution >1 nm/pixel was excluded as it had insufficient pixels for automated analysis.
Replication	Multiple molecules for each sample were imaged and each sample was replicated at least twice.
Randomization	Randomization was not carried out due to the design of the study. In both AFM and MD experiments were designed to probe a specific outcome, and therefore could not be randomized. Images were analysed using automated code to reduce bias.
Blinding	Blinding was not employed due to the unambiguous nature of the measurements, these measurements were assessed using automated code, and therefore blinding would not have reduced bias.

Reporting for specific materials, systems and methods

We require information from authors about some types of materials, experimental systems and methods used in many studies. Here, indicate whether each material, system or method listed is relevant to your study. If you are not sure if a list item applies to your research, read the appropriate section before selecting a response.

Materials & experimental systems

n/a	Involved in the study	
☒	☐	Antibodies
☒	☐	Eukaryotic cell lines
☒	☐	Palaeontology and archaeology
☒	☐	Animals and other organisms
☒	☐	Clinical data
☒	☐	Dual use research of concern
☒	☐	Plants

Methods

n/a	Involved in the study	
☒	☐	ChIP-seq
☒	☐	Flow cytometry
☒	☐	MRI-based neuroimaging

Plants

Seed stocks	N/A
Novel plant genotypes	N/A
Authentication	N/A
